# Supplementary material for: An investigation of the cognitive and neural correlates of semantic memory search related to creative ability
Source: Commun Biol. 2022 Jun 16;5:604. doi: 10.1038/s42003-022-03547-x (PMC9203494; doi:10.1038/s42003-022-03547-x)
Supplement: Supplementary file 2 — Supplementary Information [file 42003_2022_3547_MOESM2_ESM.pdf]

An investigation of the cognitive and neural correlates of semantic memory search related to creative ability

Supplementary Information

Marcela Ovando-Tellez[1], Mathias Benedek[2], Yoed N. Kenett[3], Thomas Hills[4], Sarah Bouanane[1], Matthieu Bernard[1], Joan Belo[1], Theophile Bieth[1,5] & Emmanuelle Volle[1]

[1] Sorbonne University, FrontLab at Paris Brain Institute (ICM), INSERM, CNRS, 75013, Paris, France
[2] Institute of Psychology, University of Graz, Graz, Austria
[3]Faculty of Industrial Engineering and Management, Technion – Israel Institute of Technology, Haifa 3200003 Israel
[4] Department of Psychology, University of Warwick, University Road, Coventry, CV4 7AL, United Kingdom
[5] Neurology department, Pitié-Salpêtrière hospital, AP-HP, F-75013, Paris, France

Corresponding authors:

Emmanuelle Volle

emmavolle@gmail.com

and Marcela Ovando-Tellez

marcela.ovandot@gmail.com

Supplementary Tables

Supplementary Table 1. Different meanings of the French ambiguous words. Each column has the different meanings of the three cue words used in the ambiguous word fluency task. Each word has at least five different meanings. The total number of meanings for each cue word is shown in parenthesis. A few words could not be categorized and were labeled as undetermined (< 2 % overall responses across participants).

Somme (12)	Glace (5)	Rayon (11)
amount of money	ice	department, row
French region	ice cream	radius
nap	mirror, window, looking-glass	ray, beam
to summon	emotional coldness	radiation
we are (verb to be)	icing or frosting	shelf
work		wheel spoke
summary (In sum)		honeycomb
mountain in France		domain
lexical or phonetic proximity		range
river in France		joie, light
pack animal		diverge, spread
sum (maths), amount, quantity		

Supplementary Table 2. Spearman correlations between the five measures of the PolyFT task.

		1	2	3	4	5
1	Fluency	.				
2	Rank of the first switch	.529**				
3	Number of different meanings	-.009	-.342**			
4	Number of switches	.232*	-.341**	.571**		
5	Biggest cluster size	.799**	.631**	-.367**	-.252*	.

* < .05
** < .001

Supplementary Table 3. Descriptive statistics of creativity tasks (AUT and CAT), SemNet metrics and executive tests.

	M	SD	Min	Max
Creativity Tasks				
AUT scores				
AUT-fluency	23.163	11.584	7	87
AUT-uniqueness	7.442	4.979	0	29
AUT-ratings	1.710	0.425	0.57	2.67
AUT-commonness	12.248	3.748	3.39	24.25
CAT scores				
CAT-CR	47.1	11.6	13.0	73.0
CAT-index	0.424	0.226	0	1.08
CAT-eureka	69.3	19.9	0	100
SemNet metrics				
WUN Eff	54.204	6.874	36.782	71.717
WUN CC	0.364	0.098	0.142	0.628
WUN Q	0.121	0.060	0.032	0.319
UUN Eff	0.708	0.072	0.516	0.869
UUN CC	0.586	0.082	0.438	0.781
UUN Q	0.178	0.065	0.058	0.392
Executive tests				
forward-span	11.244	2.275	6	16
backward-span	9.430	2.324	6	16
TMT-shifting	31.655	17.051	9.080	111.40
category-fluency	39.035	8.589	22	61
letter-fluency	21.361	6.473	4	35
Stroop-interference	25.143	11.019	2.810	65.990

Note. WUN= Weighted Undirected Networks; UUN= Unweighted Undirected Networks; AUT = Alternative uses task; CAT = Combination of Associates task

Supplementary Table 4. Spearman correlations between the PolyFT components, SemNet metrics for WUN and UUN graphs, creativity scores and executive functions tests. In bold are the correlations that remained significant after FDR correction for multiple comparisons. * $p < .05$; ** $p < .01$

		1	2	3	4	5	6	7	8	9	10	11	12	13	14	15	16	17	18	19	20	21
1	PolyFT clustering	.																				
2	PolyFT switching	-.15	.																			
3	AUT-fluency	**.45****	.15	.																		
4	AUT-uniqueness	**.41****	-.07	**.77****	.																	
5	AUT-ratings	.14	-.06	**.30****	**.34****	.																
6	AUT-commonness	-.27*	.07	**-.39****	**-.76****	**-.36****	.															
7	CAT-CR	-.19	.23*	-.06	-.17	.09	.19	.														
8	CAT-index	.06	-.09	-.13	-.07	-.09	.04	**-.29****	.													
9	CAT-eureka	.07	-.18	.05	.18	.08	-.12	-.16	.10	.												
10	WUN Eff	.02	.25*	-.02	.08	-.18	-.18	.13	-.04	.12	.											
11	WUN CC	.03	.16	-.06	.10	-.07	-.22*	.07	.001	.04	**.75****	.										
12	WUN Q	.08	-.07	.13	-.01	.05	.24*	-.03	.02	.09	**-.44****	**-.75****	.									
13	UUN Eff	.04	**.29****	.02	.13	-.13	-.23*	.21	-.08	.02	**.87****	**.88****	**-.64****	.								
14	UUN CC	.03	.25*	.004	.06	-.02	-.14	.12	-.01	.04	**.68****	**.75****	**-.45****	**.83****	.							
15	UUN Q	.01	-.25*	.05	-.12	.18	.20	-.07	-.02	-.09	**-.72****	**-.76****	**.81****	**-.78****	**-.55****	.						
16	forward-span	.08	.09	.27*	.20	.09	-.23*	.21*	-.11	-.13	.04	.08	-.12	.11	.02	-.09	.					
17	backward-span	.07	.23*	-.03	-.05	-.004	-.05	.09	-.03	-.17	-.06	.08	-.18	.05	.02	-.12	**.47****	.				
18	TMT-switching	-.12	-.25*	-.21	-.09	.02	.01	-.16	-.07	-.07	-.04	.04	-.10	-.01	-.09	.02	-.26*	-.18	.			
19	category-fluency	.27*	**.35****	**.36****	.23*	.08	-.04	.22*	-.18	-.07	.30**	.23*	-.12	**.32****	**.29****	**-.29****	.07	.06	- .22*	.		
20	letter-fluency	.003	**.29****	.12	.08	.11	-.03	.22*	-.03	-.05	.11	.04	.02	.10	.08	-.08	.03	.08	- .24*	**.43****	.	
21	Stroop-interference	-.06	-.26*	.03	.008	-.11	.07	-.10	-.06	.14	-.04	-.05	.08	-.03	-.02	.11	-.14	-.08	.12	-.21	-.07	.

Supplementary Figures:

Supplementary Figure 1. Functional anatomy of the negative CPM model predicting the clustering PolyFT component.

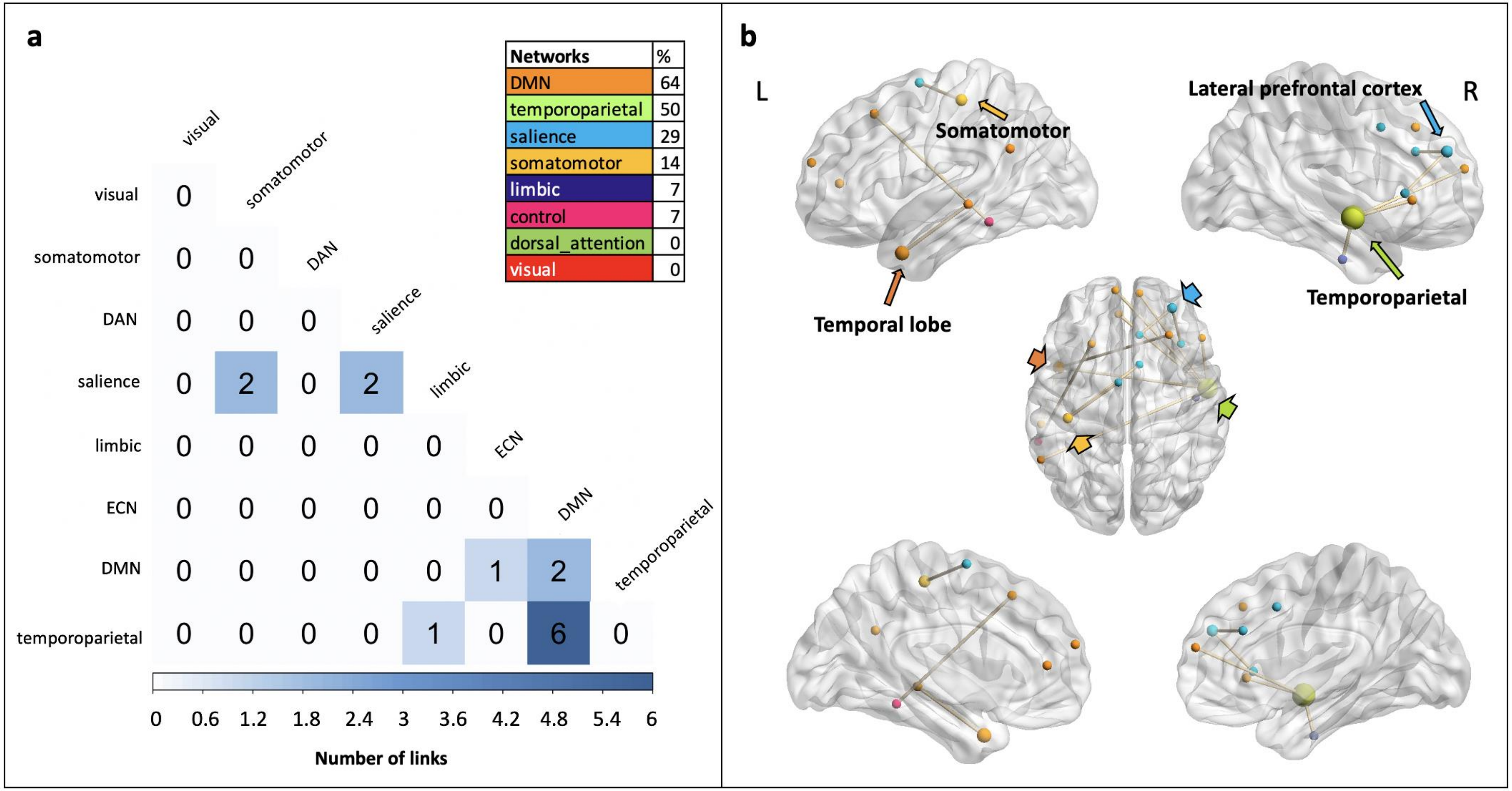

The functional connectivity patterns of the negative model network predicting clustering are described. **a** We examined the distribution of the links across intrinsic functional networks based on Schaefer's atlas [103]. The correlation matrix represents the number of links within the model network connecting within and between eight intrinsic brain networks (total of links = 14). At the upper right side, a table with the percentage of links connecting nodes that belong to these functional networks is shown. **b** The nodes (total of nodes = 18) and links of the model network are superimposed on a volume rendering of the brain. From top to bottom, lateral, dorsal and medial views for the left (L) and right (R) hemispheres are shown. The. The color of the nodes represents the functional network they belong to, using a color code presented in **a**. The size of the nodes is proportional to their degree in relation to the total number of nodes within the network. The color code arrows indicate the highest degree nodes and the brain region in which they are localized.

Supplementary Figure 2. Functional anatomy of the negative CPM model predicting the switching PolyFT component.

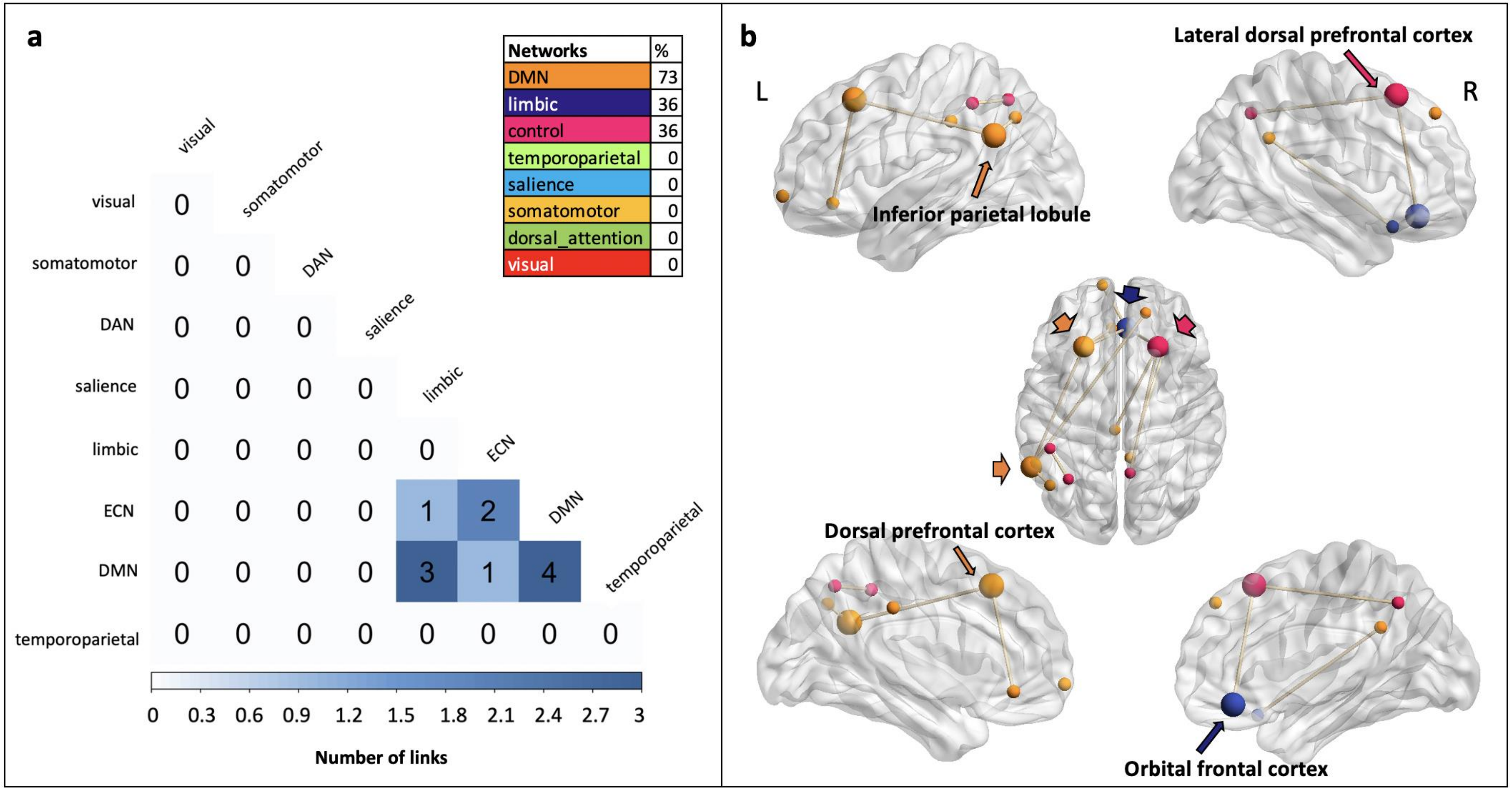

The functional connectivity patterns of the negative model network predicting switching are described. **a** We examined the distribution of the links across intrinsic functional networks based on Schaefer's atlas [103]. The correlation matrix represents the number of links within the model network

connecting within and between eight intrinsic brain networks (total of links = 11). A table with the percentage of links connecting nodes that belong to these functional networks is shown. **b** The nodes (total of nodes = 14) and links of the model network are superimposed on a volume rendering of the brain. From top to bottom, lateral, dorsal and medial views for the left (L) and right (R) hemispheres are shown. The color of the nodes represents the functional network they belong to, using a color code presented in **a**. The size of the nodes is proportional to their degree in relation to the total number of nodes within the network. The color code arrows indicate the highest degree nodes and the brain region in which they are localized.

Supplementary Figure 3. Brain connections to the temporal lobe in the CPM predictive models.

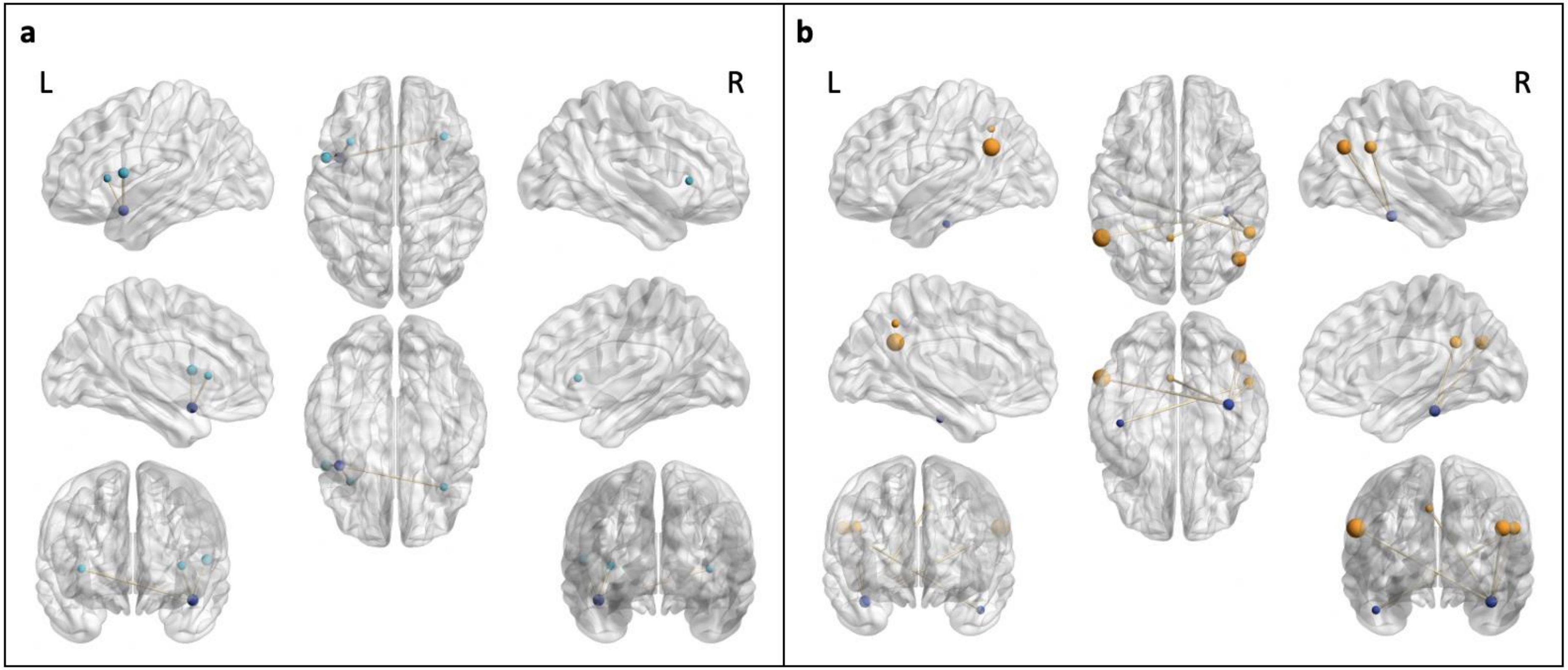

The nodes relying on the temporal lobe of the predictive model networks of clustering and switching are superimposed on a volume rendering of the brain different views for the left (L) and right (R) hemispheres. **a** In the CPM model predicting the PolyFT clustering, the left temporal pole (in purple) has connections to left and right brain regions of the salience network (in light blue). **b** In the CPM model predicting the PolyFT switching, the left and right temporal pole (in purple) have connections to left and right brain regions of the default mode network (in orange).

Supplementary notes:

Supplementary Note 1: Exploration of the area under the curve in semantic networks

We performed a data-driven analysis as a way to validate the arbitrary selection of the threshold for the UUN graphs. Based on methods proposed for brain network analysis [143], we considered a range of thresholds (35 to 65 with an interval of 5) and computed area under curve. Thus, the different network metrics (Eff, CC and Q) were integrated over a range of different thresholds to yield the area under the curve. Therefore, the statistical analyses were performing on this unique area under the curve value for each network metric. The correlation between the switching component and the UUN metrics CC (r_s = .258, *p* = .017), Q (r_s = -.231, *p* = .033) and efficiency (r_s = .269, *p* = .013) remained significant. Hence, this method led to similar results as using an arbitrary threshold of 50 (the middle of the rating scale) to select the edges in UUN.

Supplementary Note 2: Negative networks predicting clustering and switching

The negative model networks included a smaller number of links that also differed between the two components (*n* = 14 for clustering, *n* = 11 for switching). The negative model predicting clustering (**Supplementary Figure 1**) was composed of links connecting brain regions between DMN and temporoparietal networks (*n* = 6), within salience (*n* = 2), within DMN (*n* = 2) and between salience and somatomotor (*n* = 2) networks. The brain regions with the highest number of connections were located in the right temporoparietal regions of the temporoparietal network (*k* = 7), left temporal lobe of the DMN (*k* = 3), left somatomotor region of the somatomotor network (*k* = 2) and right lateral prefrontal cortex of the salience network (*k* = 2).

The negative model predicting switching (**Supplementary Figure 2**) was composed of links connecting brain regions within DMN (*n* = 4), between DMN and limbic (*n* = 3) and within ECN (*n* = 2). The brain regions with the highest number of connections were located in the right

lateral dorsal prefrontal cortex of the ECN ($k = 3$), right orbital frontal cortex of the limbic network ($k = 3$), left inferior parietal lobule ($k = 3$) and dorsal prefrontal cortex ($k = 3$) of the DMN.

Supplementary Note 3: CPM analyses controlling for motion parameters

We ran the CPM analysis without using the mean FD as a regressor in the predictive model (third step in the CPM analysis). Then, for each CPM analysis we calculated the Spearman partial correlation between the predicted and the real value of the PolyFT components, controlling for the mean FD (fourth step in the CPM analysis). The results of the Spearman partial correlations between the predicted and real values of the PolyFT components remained significant for both the clustering ($r_s = .360$, $p < .001$) and switching ($r_s = .395$, $p < .001$) components.
