# Supplementary material for: An investigation of the cognitive and neural correlates of semantic memory search related to creative ability
Source: Commun Biol. 2022 Jun 16;5:604. doi: 10.1038/s42003-022-03547-x (PMC9203494; doi:10.1038/s42003-022-03547-x)
Supplement: Supplementary file 3 — Reporting Summary [file 42003_2022_3547_MOESM3_ESM.pdf]

nature portfolio

Corresponding author(s): Marcela Ovando-Tellez and Emmanuelle Volle

Last updated by author(s): May 11, 2022

Reporting Summary

Nature Portfolio wishes to improve the reproducibility of the work that we publish. This form provides structure for consistency and transparency in reporting. For further information on Nature Portfolio policies, see our Editorial Policies and the Editorial Policy Checklist.

Statistics

For all statistical analyses, confirm that the following items are present in the figure legend, table legend, main text, or Methods section.

n/a	Confirmed	
☐	☒	The exact sample size (*n*) for each experimental group/condition, given as a discrete number and unit of measurement
☐	☒	A statement on whether measurements were taken from distinct samples or whether the same sample was measured repeatedly
☐	☒	The statistical test(s) used AND whether they are one- or two-sided<br>*Only common tests should be described solely by name; describe more complex techniques in the Methods section.*
☐	☒	A description of all covariates tested
☐	☒	A description of any assumptions or corrections, such as tests of normality and adjustment for multiple comparisons
☐	☒	A full description of the statistical parameters including central tendency (e.g. means) or other basic estimates (e.g. regression coefficient) AND variation (e.g. standard deviation) or associated estimates of uncertainty (e.g. confidence intervals)
☐	☒	For null hypothesis testing, the test statistic (e.g. *F*, *t*, *r*) with confidence intervals, effect sizes, degrees of freedom and *P* value noted<br>*Give P values as exact values whenever suitable.*
☒	☐	For Bayesian analysis, information on the choice of priors and Markov chain Monte Carlo settings
☒	☐	For hierarchical and complex designs, identification of the appropriate level for tests and full reporting of outcomes
☐	☒	Estimates of effect sizes (e.g. Cohen's *d*, Pearson's *r*), indicating how they were calculated

Our web collection on statistics for biologists contains articles on many of the points above.

Software and code

Policy information about availability of computer code

Data collection	Behavioral data were collected using custom scripts running on Matlab (Matlab R2017b, The MathWorks, Inc.,USA)
Data analysis	Analyses were conducted using open softwares and toolboxes available online as described in the Materials and Methods section (SPM 12: https://www.fil.ion.ucl.ac.uk/spm/software/spm12/; AFNI: https://afni.nimh.nih.gov; computational anatomy toolbox : CAT 12; http://dbm.neuro.uni-jena.de/cat/); Nilearn: https://nilearn.github.io/stable/index.html; TEDANA version 0.0.9 : https://tedana.readthedocs.io/en/stable/; CPM: https://www.nitrc.org/projects/bioimagesuite/; Network metrics computation: https://sites.google.com/site/bctnet/Home/functions; Brain Connectivity Toolbox.

For manuscripts utilizing custom algorithms or software that are central to the research but not yet described in published literature, software must be made available to editors and reviewers. We strongly encourage code deposition in a community repository (e.g. GitHub). See the Nature Portfolio guidelines for submitting code & software for further information.

Data

Policy information about availability of data

All manuscripts must include a data availability statement. This statement should provide the following information, where applicable:

- Accession codes, unique identifiers, or web links for publicly available datasets
- A description of any restrictions on data availability
- For clinical datasets or third party data, please ensure that the statement adheres to our policy

All data needed to evaluate the conclusions in the paper are present in the paper and/or the Supplementary Materials, or are available at https://osf.io/uktjm/?view_only=3ab90072a7804e08ad80d2b8c45ced19.

Field-specific reporting

Please select the one below that is the best fit for your research. If you are not sure, read the appropriate sections before making your selection.

☒ Life sciences ☐ Behavioural & social sciences ☐ Ecological, evolutionary & environmental sciences

For a reference copy of the document with all sections, see nature.com/documents/nr-reporting-summary-flat.pdf

Life sciences study design

All studies must disclose on these points even when the disclosure is negative.

Sample size	This is an exploratory study and effect size was not known.
Data exclusions	Data from seven participants were excluded due to a brain abnormality revealed by the MRI acquisition (n = 6), and for falling asleep during the experiment (n = 1).
Replication	leave-one -out cross-validation in statistical analyses
Randomization	no group comparison
Blinding	No blinding was done.

Reporting for specific materials, systems and methods

We require information from authors about some types of materials, experimental systems and methods used in many studies. Here, indicate whether each material, system or method listed is relevant to your study. If you are not sure if a list item applies to your research, read the appropriate section before selecting a response.

Materials & experimental systems

n/a	Involved in the study	
☒	☐	Antibodies
☒	☐	Eukaryotic cell lines
☒	☐	Palaeontology and archaeology
☒	☐	Animals and other organisms
☐	☒	Human research participants
☒	☐	Clinical data
☒	☐	Dual use research of concern

Methods

n/a	Involved in the study	
☒	☐	ChIP-seq
☒	☐	Flow cytometry
☐	☒	MRI-based neuroimaging

Human research participants

Policy information about studies involving human research participants

Population characteristics	The final sample consisted of 86 healthy participants (43 women, mean age = 25.5 years, SD = 3.48)
Recruitment	Participants were recruited via the RISC platform (https://www.risc.cnrs.fr) for online announcement
Ethics oversight	An approved French ethics committee approved the study (equivalent IRB). After being informed of the study, the participants signed a written consent form. The study was monitored by the INSERM French governmental research institute

Note that full information on the approval of the study protocol must also be provided in the manuscript.

Magnetic resonance imaging

Experimental design

Design type	task-based brain fMRI analyzed for functional connectivity and resting state
Design specifications	Participants performed six runs that consisted of 100 trials each (except for the last run with 95 trials) with 335 volumes acquired with repetition 9time (TR) = 1,600 ms. Each run was composed of four blocks with 25 trials each (except the last block that lasted 20 trials (last block of the sixth run) separated by 20 second of rest periods. An inter trial interval jittered from 0.3 to 0.7 seconds (mean 0.5; interval: 0.05) separated two successive trials.<br>A session of 15-minute resting state with a cross fixation was performed after the task.

Behavioral performance measures	In total, the participants performed 595 trials of a relatedness judgment task. Participants judged the relationships between pair of words using a visual scale ranging from 0 (unrelated words) to 100 (strongly related words). The final position of the slider in the scale after validation was considered as the semantic relatedness rating and considered for analyses.<br>Ratings were used to built weighted an unweighted semantic networks at the individual level.<br>Graph metrics (Clustering coefficient, modularity, efficiency) describing the properties of these semantic networks were used in behavioral analyses

Acquisition

Imaging type(s)	functional MRI EPI sequence and T1-weighted structural image acquired on a MRI 3T Siemens PRISMA fit TIM 64 canals
Field strength	3 Tesla
Sequence & imaging parameters	The fMRI data was acquired using multi-echo echo-planar imaging (EPI) sequences with repetition time (TR) = 1,600 ms, echo times (TE) for echo 1 = 15.2 ms, echo 2 = 37.17 ms and echo 3 = 59.14 ms, flip angle = 73°, 54 slices, slice thickness = 2.50 mm, isotropic voxel size 2.5 mm, Ipat acceleration factor = 2, multi-band = 3 and interleaved slice ordering.<br>The T1-weighted structural image was acquired using TR = 2,300 ms, TE = 2.76 ms, flip angle = 9°, 192 sagittal slices with a 1 mm thickness, isotropic voxel size 1 mm, Ipat acceleration factor = 2 and interleaved slice order.
Area of acquisition	whole brain scan

Diffusion MRI ☐ Used ☒ Not used

Preprocessing

Preprocessing software	Functional volumes of each run were first despiked, slice timing corrected and realigned to the first volume (computed on the first echo) using the afni_proc.py pipeline from the Analysis of Functional Neuroimages software (AFNI; https://afni.nimh.nih.gov). In a second step, the data were denoised using the TE-dependent analysis of multi-echo fMRI data (TEDANA; https://tedana.readthedocs.io/en/stable/), version 0.0.9. In the last step of the preprocessing, the data was co-registered on the T1-weighted structural image using the Statistical Parametric Mapping (SPM) 12 package running in Matlab (Matlab R2017b, The MathWorks, Inc.,USA).
Normalization	To spatially normalize the fMRI data, we used the transformation matrix computed from the normalization of the T1-weighted structural image, using with the default settings of the computational anatomy toolbox (CAT 12; http://dbm.neuro.uni-jena.de/cat/) implemented in SPM 12.
Normalization template	We normalized to the Montreal Neurological Institute (MNI152) template.
Noise and artifact removal	The data were denoised using the TE-dependent analysis of multi-echo fMRI data (TEDANA; https://tedana.readthedocs.io/en/stable/), version 0.0.9. The TEDANA pipeline consisted of an optimal combination of the echo time series followed by the reduction of the data using PCA and independent component analysis (ICA) to decompose the multi-echo BOLD data, and classify the BOLD components as BOLD or non-BOLD. The removal of the latter eliminates the thermal and physiological noise including the artefacts generated by the movements, respiration, and cardiac activity.<br>We covaried out the task-related signal from each run, the denoised and normalized fMRI data were entered in a general linear model in SPM. We regressed out of the BOLD signal 24 motion parameters (standard motion parameters, first temporal derivatives, standard motion parameters squared, and first temporal derivatives squared) and the onsets and durations of each task related events (reflection period, response period, inter trial interval, cross fixation periods, and change of the cross-fixation colour). We then standardized and detrended the residuals of the GLM for each run, and concatenated the six runs, removing the between runs rest periods. This preprocessed data was used in the CPM analyses.
Volume censoring	No volume was discarded from any of the fMRI data since the recording did not contain dummy scans.

Statistical modeling & inference

Model type and settings	Connectome-based predictive modeling based on functional connectivity<br>Leave-one-out validation<br>Permutation for prediction testing
Effect(s) tested	Linear model of brain connectivity predicting our behavioral measures (clustering and switching component) allowed us to build the model that was tested on the left-out participant

Specify type of analysis: ☐ Whole brain ☐ ROI-based ☒ Both

Anatomical location(s)	We defined regions of interest (ROIs) based on the Schaefer brain atlas which includes 200 ROIs of 2-mm dimensions distributed into 17 functional subnetworks distributed on eight main functional networks. Individual functional connectivity matrices were built using these ROIs.<br>The functional connectivity matrix of each participant was computed using Nilearn v0.3 (Abraham et al., 2014) in Python 2.7 (van Rossum, 1995). We defined regions of interest (ROIs) based on the Schaefer brain atlas (Schaefer et al., 2018) which includes 200 ROIs of 2-mm dimensions distributed into 17 functional subnetworks distributed on eight main functional networks. We extracted and averaged the BOLD signal for each ROI and performed Pearson correlations pairwise. As a result, we obtained a 200 x 200 matrix for each participant corresponding to the functional connectivity network of each participant in which ROIs are the nodes and correlation coefficients the links. These matrices were Z-Fisher transformed and rescaled by the maximal weight to be in the range of -1 to 1 for the subsequent analyses.
Statistic type for inference (See Eklund et al. 2016)	atlas-based
Correction	Behavioral data were FDR corrected. Functional connectivity resuts using the CPM method used a leave-one-out cross-validation approach and corrected using permutations

Models & analysis

n/a	Involved in the study
☐	☒ Functional and/or effective connectivity
☐	☒ Graph analysis
☐	☒ Multivariate modeling or predictive analysis

Functional and/or effective connectivity	Pearson correlation
Graph analysis	Graph analysis was only used in the behavioral part of the study, to explore semantic networks.
Multivariate modeling and predictive analysis	We performed a connectome based predictive modeling (Shen et al., 2017). We used a leave one out cross validation: 1. We selected the connections in the functional connectivity matrix (z-scored connectivity values between ROIs) that significantly correlated with our behavioral measure (threshold p < .01) either positively or negatively across participants (N-1). We used Spearman correlations to avoid the possible influence of outliers in the predictions. 2. we estimated the connectivity strength in these model networks for each participant by summing the functional connectivity values of the selected ROI pairs. 3. We built a linear model with the resulting individual connectivity strength in the positive and negative model networks as predictors and the behavioral measure as the outcome. 4. We applied iteratively the predictive linear model built on N-1 participants to the left-out participant. 5. We tested the prediction of the linear model by running Spearman correlations between the predicted and the observed values. 6. we evaluated the predictive power of the predictions using permutation testing. Finally, we validated our predictive model built in the on-task data, by testing this model in the resting state data.
